# A Novel Role of Interleukin 13 Receptor alpha2 in Perineural Invasion and its Association with Poor Prognosis of Patients with Pancreatic Ductal Adenocarcinoma †

**DOI:** 10.3390/cancers12051294

**Published:** 2020-05-20

**Authors:** Toshio Fujisawa, Takeshi Shimamura, Kaku Goto, Ryo Nakagawa, Ryosuke Muroyama, Yoshinori Ino, Hajime Horiuchi, Itaru Endo, Shin Maeda, Yasushi Harihara, Atsushi Nakajima, Nobuyuki Matsuhashi, Naoya Kato, Hiroyuki Isayama, Ankit Puri, Akiko Suzuki, Ian Bellayr, Pamela Leland, Bharat H. Joshi, Raj K. Puri

**Affiliations:** 1 Department of Gastroenterology, Juntendo University, Tokyo 113-8421, Japan; t-fujisawa@juntendo.ac.jp (T.F.); h-isayama@juntendo.ac.jp (H.I.); 2 Division of Advanced Genome Medicine, Advanced Clinical Research Center, The Institute of Medical Science, The University of Tokyo, Tokyo 108-8639, Japan; kgoto@unistra.fr (K.G.); Ryo420@chiba-u.jp (R.N.); Muroyama-dm@umin.org (R.M.); kato.naoya@chiba-u.jp (N.K.); 3 Tumor Vaccines and Biotechnology Branch, Division of Cellular and Gene Therapies, Center for Biologics Evaluation and Research, Food and Drug Administration, Silver Spring, MD 20993, USA; Ankit.puri@fda.hhs.gov (A.P.); akikosuzuki2009@gmail.com (A.S.); ian.bellayr@nih.gov (I.B.); pjdover12@gmail.com (P.L.); 4 Department of Gastroenterology and Hepatology, Yokohama City University School of Medicine, Yokohama 236-0004, Japan; takesuica@ip-members.net (T.S.); nakajima-tky@umin.ac.jp (A.N.); 5 Pathology Division, National Cancer Center Research Institute, Tokyo 104-0045, Japan; tossshy@ybb.ne.jp; 6 Department of Pathology, NTT Medical Center, Tokyo 141-0022, Japan; horiuchi-path@umin.ac.jp; 7 Department of Gastroenterological Surgery, Yokohama City University School of Medicine, Yokohama 236-0004, Japan; endoit@med.yokohama-cu.ac.jp; 8 Department of Gastroenterology, Yokohama City University School of Medicine, Yokohama 236-0004, Japan; smaeda@med.yokohama-cu.ac.jp; 9 Department of Surgery, NTT Medical Center, Tokyo 141-0022, Japan; harihara-tky@umin.ac.jp; 10 Department of Gastroenterology, NTT Medical Center, Tokyo 141-0022, Japan; matuhasi@east.ntt.co.jp

**Keywords:** IL-13Rα2, pancreatic ductal adenocarcinoma, prognosis, perineural invasion

## Abstract

Perineural invasion (PNI) is one of the major pathological characteristics of pancreatic ductal adeno-carcinoma (PDAC), which is mediated by invading cancer cells into nerve cells. Herein, we identify the overexpression of Interleukin-13 Receptor alpha2 (IL-13Rα2) in the PNI from 236 PDAC samples by studying its expression at the protein levels by immunohistochemistry (IHC) and the RNA level by in situ hybridization (ISH). We observe that ≥75% samples overexpressed IL-13Rα2 by IHC and ISH in grade 2 and 3 tumors, while ≥64% stage II and III tumors overexpressed IL-13Rα2 (≥2+). Interestingly, ≥36 % peripancreatic neural plexus (PL) and ≥70% nerve endings (Ne) among PNI in PDAC samples showed higher levels of IL-13Rα2 (≥2+). IL-13Rα2 +ve PL and Ne subjects survived significantly less than IL-13Rα2 –ve subjects, suggesting that IL-13Rα2 may have a unique role as a biomarker of PNI-aggressiveness. Importantly, IL-13Rα2 may be a therapeutic target for intervention, which might not only prolong patient survival but also help alleviate pain attributed to perineural invasion. Our study uncovers a novel role of IL-13Rα2 in PNI as a key factor of the disease severity, thus revealing a therapeutically targetable option for PDAC and to facilitate PNI-associated pain management.

## 1. Introduction

Pancreatic ductal adenocarcinoma (PDAC) is identified as one of the highly aggressive cancers, with increased mortality among gastrointestinal cancer patients [1]. A complete surgical intervention of highly localized pancreatic cancer may lead to a complete cure from this deadly disease. However, it is commonly seen that the pancreatic cancer is characterized by the local spread to adjacent tissues or organs, with early metastatic lesions to the nearest lymph nodes, liver, and surrounding nerves within the pancreas and the peripheral nerve plexus [2]. Most cases of pancreatic cancer present with local invasion of principal arteries and metastasis to the liver at the time of diagnosis. Therefore, only 20% of cases have attained the five-year survival rate of <20% after a complete surgical resection [3,4].

A potential reason for tumor recurrence may be due to its highly invasive characteristic of attacking the surrounding nerves in a process termed perineural invasion (PNI). PDAC is associated with one of the highest incidences of PNI among various human cancers, and its presence in PDAC is believed to be a biomarker of poor prognosis [5,6]. Furthermore, the higher expression of the transforming growth factor-α (TGFα) within neurons adjacent to the pancreas and the increased expression of epidermal growth factor receptor (EGFR) on the pancreatic cancer cells correspond to an increased affinity of neurons for pancreatic cancer cells or vice versa [7]. Finally, it has been demonstrated that nerve growth factors (NGF) produced by tumor-associated immune cells and fibroblasts can be causative factors for pain generation through binding with tropomyosin receptor kinase A (*TrkA*) and neurotrophin receptor *p75*(P75^NTR^) to trigger neurogenic inflammation [8,9], which are overexpressed in pancreatic cancer cells and adjacent nerves and not typically seen in normal exocrine pancreas [10,11]. The expression of NGF and *TrkA* are significantly correlated with the incidence of PNI [10]. Like NGF, the nerve-released glial cell line-derived neutrotrophic factor (GDNF) family receptor (GFR) α1 is identified as one of the key factors involved in PNI through GDNF-Ret proto-oncogene (RET) signal transduction [12].

IL-13Rα2 is a high affinity receptor binding protein to Th2 derived cytokine IL-13 and identified as a cancer testis antigen [13,14]. We have demonstrated that IL-13Rα2 is overexpressed in numerous solid human cancers such as malignant glioma, squamous cell carcinoma of head and neck, Kaposi’s sarcoma, kidney cancer, adrenocortical cancer, and ovarian carcinoma [15,16] and that IL-13Rα2 can be efficiently targeted by a chimeric recombinant protein, which consists of IL-13 and truncated Pseudomonas exotoxin [17,18]. In contrast, normal immune cells do not express or weakly express this receptor chain [19,20]. Pancreatic cancer also overexpresses moderate to high-density IL-13Rα2 in about 70% of the samples [21]. Our previous study in human glioma cell lines revealed that there was no mutation identified in IL-13Rα2 cDNA and its promoter by polymerase chain reaction (PCR)-based single-strand conformation polymorphism studies [22]. No such mutations have been identified and reported yet in PDAC tumors.

The expression of IL-13R, its composition and crosstalk have been extensively studied in our laboratory [15,16]. We and others have shown that IL-13 binds to two different receptor chains, IL-13Rα1 and IL-13Rα2. IL-13 binds to IL-13Rα1 with low affinity while it binds to IL-13Rα2 with high affinity. IL-13Rα2 is uniquely expressed in cancer cells, diseased fibroblasts, and macrophages. For signal transduction, IL-13 binds to IL-13Rα1 with a low affinity which then recruits IL-4Rα chain to form a heterodimer high affinity complex and mediate signaling through Janus kinase/signal transducers and activators of transcription JAK/STAT pathway mediating proliferation and other functions of normal immune cells [15,16,23,24]. On the other hand, IL-13 when bound to IL-13Rα2 in tumor cells does not recruit another chain but mediates signaling through a different pathway. IL-13 binds to IL-13Rα2 and activates activation protein 1(AP-1) and extracellular Signal-Regulated Kinase 1 and 2 (ERK1/2) followed by induction of transforming growth factor (TGF) β, resulting in increased metastasis of tumors [21,25,26]. Interestingly, IL-13Rα1, which is not overexpressed in great abundance compared to IL-13Rα2 in solid tumors, is another binding protein to its ligand IL-13 that has recently been observed to co-exist with Kirsten rat sarcoma KRAS in pancreatic cancer cells supporting a pro-tumorigenic tumor microenvironment [27]. However, the mechanism of overexpression of IL-13Rα2 and its role in human cancer is not clear and under investigation. In pancreatic cancer, IL-13 can signal after binding to IL-13Rα2 via the AP-1 pathway, and IL-13Rα2 expression is dependent upon histone acetylation [21,28]. We have also demonstrated that IL-13 can enhance pancreatic cancer invasion and metastasis after binding to IL-13Rα2 via the upregulation of extracellular-signal-regulated kinase (ERK) and Matrix metallo-proteinases (MMPs) in the murine orthotopic pancreatic cancer model [29]. Other investigators have reported that IL-13Rα2 gene expression is significantly increased in metastatic lesions in the lungs of patients with breast cancer [30].

In the present study, we investigate IL-13Rα2 expression and its association with neural invasion in human PDAC samples. Among ten clinicopathological factors of PDAC, our findings uniquely demonstrate that IL-13Rα2 is significantly overexpressed in the peripancreatic neural plexus and nerve endings, which is correlated with the poor survival of patients. As PNI is associated with the generation of pain experienced by the PDAC patients and associated with IL-13Rα2 overexpression, we hypothesize that IL-13Rα2-targeted therapy may be a useful and potent approach to inhibiting cancer invasion, metastasis and PNI, hence, it may prolong the survival of PDAC patients and alleviate their pain.

## 2. Results

### 2.1. Demographic and Clinicopathological Correlates of Tumor Samples at Presentation in Patients with PDAC

Two PDAC sample sets derived from two different institutions were combined and independently evaluated for IL-13Rα2 expression and correlated with clinical data. The demographic information of patients from the two hospitals are shown in Table 1. Eighty of 236 patients (34.3%) had well differentiated, 55.5% moderately differentiated, and 10.2% poorly differentiated disease. Among these, 12% patients presented with clinical stage I, 38% with stage II, 36% with stage III and 14% with stage IV disease. There were 63% male and 37% female patients. These patients had different clinicopathological presentations of cancer invasion and survived 9–17 months with poorly differentiated grades and 18–29 months with well-to-moderately differentiated grades (Table 2). The patients with invasive disease to PL (peripancreatic neural plexus) and Ne (nerve endings) survived relatively shorter than those with well or moderately differentiated disease.

The demographic analysis of 236 patients from two hospitals was performed with grades and stages of the disease.

### 2.2. IHC Results of IL-13Rα2 Expression in PDAC Correlate with Pathologic Grade and Clinical Stage of PDAC

**Figure 1:**
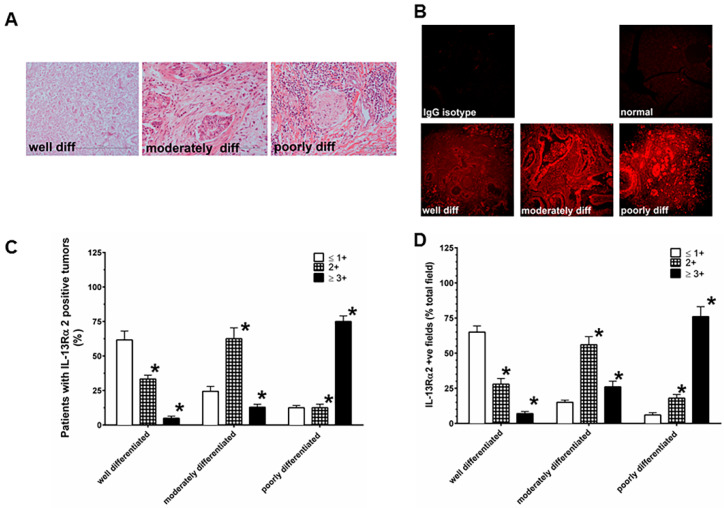
PDAC (pancreatic ductal adeno-carcinoma) samples are characterized by IL-13Rα2 expressing tumor cells in different pathological grades. (**A**) H&E (hematoxylin and eosin) staining of PDAC samples with well differentiated, moderately differentiated, and poorly differentiated tumors. (**B**) Immunohistochemical analysis for IL-13Rα2 expression in PDAC and normal pancreas. (**C**) The extent of immunostaining of IL-13Rα2 in PDAC was evaluated at three levels between 0 and 3+ according to the intensity of staining. (**D**) Percent positive fields expressing IL-13Rα2 were counted in samples with different pathological grades. Normal pancreas showed negative staining for IL-13Rα2 expression. The samples were viewed at 200× magnification. * *p* = 0.0001.

We examined the expression of IL-13Rα2 in 236 PDAC samples with different pathological grades and clinical stages. Typical hematoxylin and eosin (H&E) and immunofluorescence immunostaining patterns for IL-13Rα2 expression in pancreatic cancer samples are shown in Figure 1A,B. Further, we examined the IL-13Rα2 subunit expression in 236 PDAC samples from different pathological grade and clinical stage databases. The intensity of IL-13Rα2 subunit expression by immunohisto-chemistry (IHC) was analyzed by six investigators in a blinded fashion. To assess the precision of the pathological grade database, 236 PDAC samples were evaluated by six investigators on two different occasions, resulting in twelve different sets of readings to score IL- 13Rα2 immunostaining intensity. Similarly, 236 PDAC samples in the clinical stage database were also examined, which generated twelve independent readings for each slide. Immunostaining intensity scores of ≥2+ were considered positive, while ≤1+ were considered negative. A very high concordance among these twelve sets of readings was observed in both databases, where scores 3+ and 4+ were combined as 3+, while scores 1+ and no score (≤1+) were combined as 1+. Of the samples, 214/236 (90.6%) showed concordant scores (exact 95% confidence interval CI = 76.8–96.6) for the pathological grade database, and 202/230 (87.8%) samples demonstrated concordant scores (exact 95% CI = 71.2–92.1) for clinical stage database. Our results exhibited high precision in the IHC for both sets of databases. For the remainder of the data analysis, the discordant score values were determined by assigning the majority score, and when there was an equal number of discordant scores, the lower score was considered. For the rest of the analysis, all discordant cases were considered as negative for IL-13Rα2 expression. We calculated median ± standard deviation (SD) of these twelve sets to form a final score. These results showed high precision for both databases.

As shown in Figure 1, IHC results show that the extent of immunostaining for IL-13Rα2 is significantly higher as the grade advances from well differentiated to moderately to poorly differentiated PDAC tumor samples (Figure 1A,B). The combined positive values of IL-13Rα2 (2+ and 3+) immunostaining intensity showed a statistically significant higher trend with the pathological grade of the disease (well differentiated = 38.3%, moderately differentiated = 75.6%, poorly differentiated = 87.5%; exact *p* < 0.0001) in comparison with negative values of IL-13Rα2 (≤1+) (Figure 1C). Similarly, a significantly increasing trend was also detected even after adjusting for age (*p* = 0.0025) in this cohort (Fujisawa et al, Bethesda, MD, USA; data not shown). In contrast, 12 normal pancreas specimens showed ≤1+ staining for IL-13Rα2 (Figure 1B). Similarly, the percentage of positive fields for IL-13Rα2 expression in 2+ and 3+ positive specimens in IHC analysis demonstrated a highly significant trend in these specimens (well differentiated = 35.0%, moderately differentiated = 82.0% and poorly differentiated = 94%; exact *p* < 0.0001) compared to PDAC samples with negative values of IL-13Rα2 (≤ 1+) (Figure 1D). We observed that only tumor cells showed appreciable IL-13Rα2 immunostaining. It is possible that IL-13R α2 is also expressed in stroma and immune cells, however, their staining appears below the detection limits of the IHC and in situ hybridization (ISH) techniques used in the present study. A majority of the tumor cells have membranous and cytoplasmic immunostaining.

**Figure 2:**
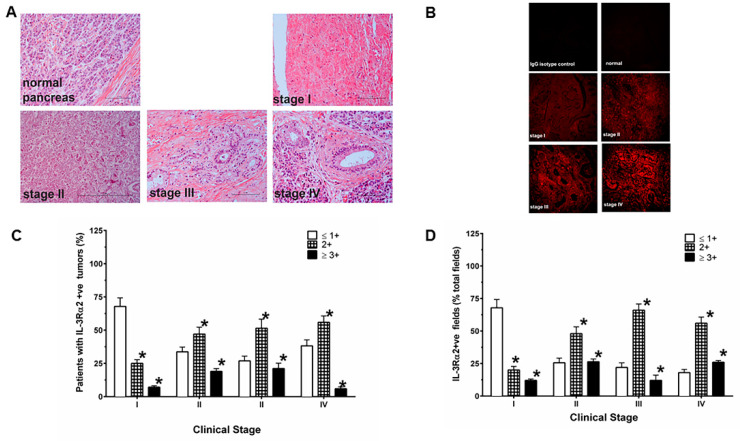
IL-13Rα2 expressing tumor cells in different clinical stages. (**A**) H&E staining of PDAC samples with stage I-IV tumors (**B**) IHC (immunohisto-chemistry) of IL-13Rα2 expression in PDAC with different stages and normal pancreas. (**C**) The extent of IHC staining in PDAC was evaluated at four levels between 0 and 3+ according to the intensity of immunostaining. (**D**) Percent positive fields expressing IL-13Rα2 were counted in samples with different grades. Normal pancreas showed negative staining for IL-13R2e expression. The samples were viewed at 200× magnification. * *p* = 0.0001.

PDAC samples with different clinical stages revealed a significant increase in the proportion of patients as the stage advanced from stage I to III–IV for the intensity of immunostaining of IL-13Rα2 expression (Figure 2A,B). Interestingly, the combined IHC positive values for IL-13Rα2 (≥2+ and 3+ combined) in comparison to samples with negative values (≤1+) showed a statistically significant increasing trend with the clinical stages (stage I = 32.1%, stage II = 66.3.2%, stages III–IV = 69.7%; exact *p* ≤ 0.0001). This pattern of IL-13Rα2 expression was also observed even after adjusting for age and gender (*p* = 0.002, Fujisawa et al, Bethesda, MD, USA; data not shown). Twelve normal pancreas specimens showed ≤1+ staining for IL-13Rα2 (Figure 2C). Similarly, the percentage of positive fields for IL-13Rα2 expression in 2+ and 3+ positive specimens in IHC analysis demonstrated a highly significant trend in these specimens with different stages of disease (stage I = 21.4%, stage II = 47.2% and stage III = 84.9%; exact *p* < 0.0001) compared to negative values of IL-13Rα2 (≤1+) (Figure 2D).

### 2.3. Expression of IL-13Rα2 mRNA by ISH in PDAC Correlates with Pathologic Grade and Clinical Stage

As shown in the supplementary figure (SI), Figure S1A–D, ISH data reveal that the levels of IL-13Rα2 mRNA also showed a significant increase in hybridization intensity for IL-13Rα2 mRNA (supplementary Figure S1B), with the well differentiated to moderately to poorly differentiated grades (H&E images shown in supplementary figure (Figure S1A) corroborating the IHC analysis. The total number of samples with positive hybridization for IL-13Rα2 (2+ and 3+) also demonstrated a statistically significant increasing pattern with the pathologic grade (well differentiated = 50.6%, moderately differentiated = 82.4%, poorly differentiated = 79.2%; exact *p* < 0.00014) compared to PDAC tumors with negative values of IL-13R**α**2 (≤1+) (SI Figure S1C). The significant increasing trend in PDAC samples was also observed even after adjusting for age (*p* ≤ 0.0022) in this cohort (Fujisawa et al, Bethesda, MD, USA; data not shown). In contrast, 12 normal pancreas specimens showed ≤1+ intensity for hybridization for IL-13Rα2 mRNA. Similarly, the percentage of positive fields for IL-13Rα2 mRNA in 2+ and 3+ positive specimen in ISH analysis demonstrated a highly significant trend in these specimen (well differentiated = 47.2%, moderately differentiated = 91.2% and poorly differentiated = 95.4%; exact *p* < 0.00011) compared to samples with negative values of IL-13Rα2 (≤ 1+) (supplementary figure, Figure S1D).

PDAC samples with different clinical stages confirmed a significant rise in IL-13Rα2 mRNA in samples as the stages progressed from stages I to III–IV for ISH intensity in the supplementary figure (Figure S2B). H&E staining of these samples is shown in Figure S2A. Interestingly, the intensity of hybridization for positive values of IL-13Rα2 (2+ and 3+ combined), when compared to samples with negative values of IL-13Rα2 (≤1+), showed a statistically significant increasing pattern with the stage of disease (stage I = 50.0 %, stage II = 69.7 %, stages III–IV = 77.3 %; exact *p* ≤ 0.0001) (Figure S2C). This trend was continued even after adjusting for age and gender (*p* = 0.002, Fujisawa et al, Bethesda, MD, USA; data not shown). Similarly, the percentage of positive fields for IL-13Rα2 mRNA expression in 2+ and 3+ positive specimens in ISH analysis demonstrated a highly significant trend in these specimen (stage I = 21.4%, stage II = 47.2%, stages III-IV = 84.9%; exact *p* < 0.00011) compared to samples with negative values of IL-13R**α**2 (≤ 1+) (Figure S2D).

### 2.4. Heterogeneity in IL-13Rα2 Expression is Associated with Clinicopathological Attributes of PDAC

A total of 69 (29.5%) and 160 (67.7%) of 236 PDAC specimen with a moderately to poorly differentiated pathological grade, respectively, were detected with invasion of the PL and Ne as evident by H&E staining. The remainder of the samples from the PL and Ne groups, 167/236 (70.5) and 76/236 (32.3 %) were identified as well differentiated pathological grade samples (Table 2).

We then stratified the data based on IHC immunostaining for IL-13Rα2 positivity, which revealed that 56 (36%) samples demonstrated IL-13Rα2 positive tumor cells that had invaded PL and were of moderate to poor pathological grade. We observed that 100 (64%) specimen with IL-13Rα2 positive immunostaining did not show PL invasion and were of a well-differentiated pathologic grade. Interestingly, 13 (16%) PL invasion positive samples with a moderate to poor grade also revealed IL-13Rα2 positive immunostaining. The remaining 67 (84%) samples with no invasion of the PL and no IL-13Rα2 positive immunostaining were well-differentiated.

In contrast, the number for IL-13Rα2 positive PDAC specimens rose to 117 (75%) with Ne invasion while 39 (25%) samples with no Ne− invasion were positive for IL-13Rα2 (*p* ≤ 0.01; Table 3). The number of IL-13Rα2 negative and Ne positive PDAC was 43 (54%) while 37 (46%) were negative for both IL-13Rα2 and Ne. Furthermore, the samples with no invasion of Ne were well differentiated, which is similar to the samples with no invasion to PL. Excluding patient survival, IL-13Rα2 expression strongly (correlation index >0.2) and significantly (*p*-value < 0.05) correlated with invasion to the peripancreatic neuroplexus (PL), the back constructs of pancreas (RP), and nerve in the pancreas (Ne). These two parameters that were invasion to peripancreatic neuroplexus (PL) and nerve (Ne), were correlated with IL-13Rα2 expression in terms of patient survival.

### 2.5. Association of IL-13Rα2 with Perineural Invasion

We analyzed the association of IL-13Rα2 expression with perineural invasion of the pancreatic cancer. IL-13Rα2-positive cancers showed 36% (56/156) invasion of peripancreatic neuroplexus (PL), while IL-13Rα2-negative cancer showed only 16% (13/80) in the combined data from both hospitals (Table 3). Similarly, IL-13Rα2-positive cancer samples showed 75% (117/156) Ne, but IL-13Rα2-negative cancer showed only 54% (54/80). The number of PL and Ne positive PDAC were found to be significantly higher in the IL-13Rα2-positive cancers. Interestingly, a greater number of tumor samples with invasion to Ne rather than PL were significantly associated with enhanced IL-13Rα2 detected by IHC and mRNA (ISH) (*P* ≤ 0.0001) in PDAC patients with a moderate to poor pathologic grade.

### 2.6. IL-13Rα2 is Associated with Overall Survival of PDAC Patients

**Figure 3:**
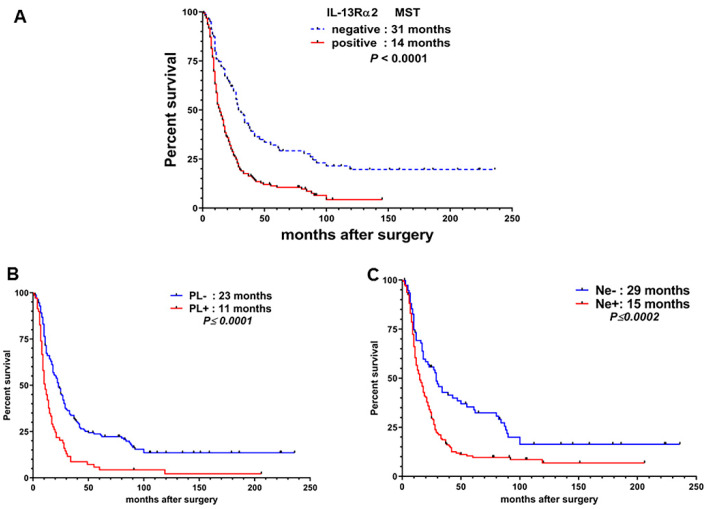
Kaplan–Meier survival curve of patients after pancreatic cancer resection. Survivals were compared between patients with (**A**) IL-13Rα2-positive tumors and IL-13Rα2-negative tumors, (**B**) PL– and PL+ and, (**C**) Ne– and Ne + tumors.

Next, we performed Kaplan–Meier survival analysis of all 236 PDAC patients, which revealed that the median survival time (MST) of 80 patients with IL-13Rα2-negative tumors were 31 months compared to 14 months in the 156 IL-13Rα2-positive PDAC patients (Figure 3A). Log-rank test analysis revealed that the survival time of patients with IL-13Rα2-positive tumors was significantly shorter than patients with negative tumors in the combined data from NTT (東日本関東病院) and Yokohama City University YCU hospitals (HR; 2.214, 95% CI; 1.475–2.954, *p* < 0.0001). We sub-stratified these results for patients with PL−/PL+ and Ne−/Ne+ diagnosis. As shown in Figure 3B, the MST for PL− patients were 23 months compared to 11 months in PL+ patients (*p* ≤ 0.0001); and 29 months for Ne− and 15 months in Ne+ patients (*p* ≤ 0.0002; Figure 3C).

### 2.7. Analysis of Clinicopathological Findings and Prognostic Factors by Multivariate Analysis

The clinicopathological parameters were investigated using the log-rank test for relationship to patient survival in the combined dataset (Table 2). Fifteen parameters including tumor size, differentiation, invasion of bile duct (CH), to duodenum (DU), to front constructs of pancreas (S), to back constructs of pancreas (RP), to portal vein (PV), to artery (A), to peripancreatic neuroplexus (PL), to the other organs (OO), to lymph duct in the pancreas (Ly), to vein in the pancreas (V), to nerve in the pancreas (Ne), Union Internationale Contre le Cancer (UICC)-stage, and CA19-9, significantly affected patient survival on univariate analysis. Sixteen parameters that included IL-13Rα2 expression and 15 clinicopathological parameters were also analyzed by multivariate analysis by Cox’s proportional hazards model. Only four parameters including IL-13Rα2 expression, UICC-clinical stage, CA19-9, and invasion to front constructs of pancreas (S) were independent prognostic factors on multivariate analysis (Table 2). In addition, an extensive analysis to determine any correlation between adjuvant chemotherapy and IL-13 Rα2 expression or prognosis of these patients was performed, however, no correlation between IL-13Rα2 expression and adjuvant therapy was observed. Furthermore, none of the subjects included in this study had received neoadjuvant chemotherapy (Fujisawa et al, Bethesda, MD, USA; data not shown).

Ten parameters significantly affected patient survival at the NTT hospital while 14 parameters affected patient survival at YCU hospital by univariate analysis. Multivariate analysis revealed that only four parameters including IL-13Rα2 expression, UICC-stage, tumor differentiation, and invasion of lymph duct (Ly), and three parameters that included IL-13Rα2 expression, UICC-stage, and CA19-9 were independent prognostic factors (Table 1). IL-13Rα2 expression and UICC-stages were found to be common prognostic factors in the combined data from the two hospitals by multivariate analysis.

### 2.8. IL-13Rα2 in PNI is Postively Correlated with Decreased Survival in PDAC Patients

**Figure 4:**
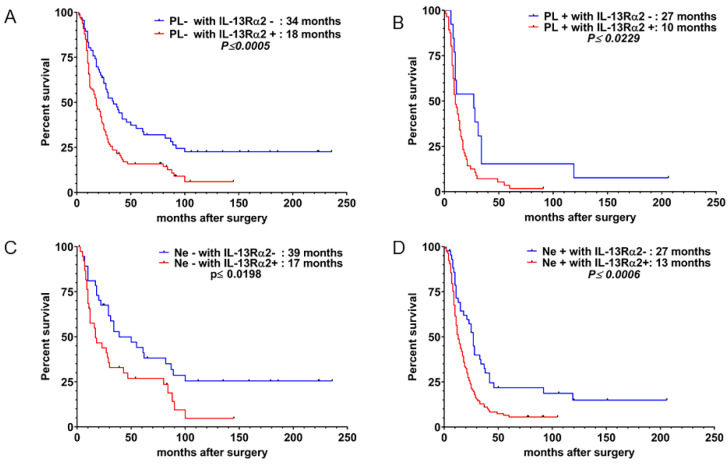
Kaplan–Meier survival curve of patients after pancreatic cancer resection. Survivals were compared between patients with (**A**) PL− with IL-13Rα2− vs. PL− with IL-13Rα2+,(**B**) PL+ with IL-13Rα2− vs. PL+/ IL-13Rα2+ (**C**) Ne− with IL-13Rα2− versus Ne− with /IL-13Rα2+; and (**D**) /Ne+ with IL-13Rα2– vs. Ne+ with IL-13Rα2+ PDAC tumors.

To evaluate the usefulness of IL-13Rα2 as an index of monitoring therapy and the natural history of PDAC patients, we further stratified and sub-categorized the results into four groups, each with PL− with IL-13Rα2−, PL– with IL-13Rα2+ (Figure 4A), PL+ with IL-13Rα2− and PL+ with IL-13Rα2+ (Figure 4B) and studied their MST. We observed that the MST for PL− plus IL-13Rα2− patients is significantly higher than that of PL− with IL-13Rα2+ (34 months vs. 18 months, *p* ≤ 0.0005). As shown in Figure 4B, PL+ with IL-13R**α**2− patients lived 17 months longer when compared to 10 months for PL+ with IL-13Rα2+ patients (*p* ≤ 0.0229). Similarly, we observed a significant increase in survival time in patients with Ne− with IL-13Rα2− versus Ne− with IL-13Rα2+ (Figure 4C, *p* ≤ 0.0198) and Ne+ with IL-13Rα2− vs. Ne+ with IL-13Rα2+ patients (Figure 4D, *p* ≤ 0.0006).

## 3. Discussion

We have previously reported that approximately 70% of pancreatic cancers express moderate to high-density IL-13Rα2 [21]. Our present results also show a similar trend and demonstrate that 66% of surgically resected pancreatic cancer samples obtained from NTT and YCU hospitals expressed IL-13Rα2. On the basis of these results, we believe that IL-13Rα2 expression in surgically resected PDAC is a biomarker that requires further evaluation. Because the heterogeneity of IL-13Rα2 expression in pancreatic cancer is minimal and the concordance among investigators evaluating the intensity by IHC is high, even a small sample collected by endoscopic ultrasound-guided fine needle aspiration (EUS-FNA) technique may reflect the IL-13Rα2 expression of the entire tumor. An evaluation of the IL-13Rα2 level on the Endoscopic ultrasound/fine needle aspiration (EUS) EUS-FNA sample might be useful for predicting not only the effectiveness of the anti-cancer therapy targeting IL-13Rα2 but also the patient’s prognosis, as substantial EUS-FNA or fine needle biopsy (FNB) samples collected at both hospitals have enabled accurate diagnosis of ~100% for common invasive cancers. In addition, EUS-FNA technology has enabled the execution of a gene panel diagnosis of PDAC.

In our study, the pancreatic cancer samples from two different hospitals (NTT and YCU) were examined to confirm the reproducibility of these results. Our data demonstrate that IL-13Rα2 expression correlates with the severity and disease aggressiveness in terms of pathologic grade and clinical stage of the patients. Furthermore, our observations of a higher incidence of IL-13Rα2 positive tumor cells invading peripancreatic neuroplexus and nerve endings in advanced pathologic grades and clinical stages may suggest their novel role as a biomarker of disease pathogenesis, cancer invasion, and metastasis through PNI in PDAC. Only two factors, IL-13Rα2 expression and UICC-stage, were common prognostic factors on multivariate analysis, which indicate that IL-13Rα2 expression may be an equally strong prognostic factor similar to clinical staging of disease (UICC-stage).

Interestingly, by correlation coefficient analysis, pancreatic cancer invasion to the neuroplexus and nerve strongly correlated with IL-13Rα2 expression in tumors obtained from both hospitals. Thus, it appears that IL-13Rα2 expression of tumors and nerves may be a highly coordinated process involving multiple signaling events initiated by both PDAC cells and nerves, generating a mutual tropism between them. To the best of our knowledge, we believe these results are novel and are the first to demonstrate a role for IL-13Rα2 in neural invasion in PDAC that can progress to distant metastatic lesions. Similarly, IL-13Rα2 overexpression in tumors facilitates the invasion of the nervous system for developing metastatic lesions, which is a topic of ongoing research in our laboratory. Based on our previous studies, IL-13Rα2 has a critical role in tumor invasion and metastasis. Our present study suggests that overexpression of IL-13Rα2 is associated with increased severity of the disease in terms of advanced stage disease and pathological grade in a relatively large sample size of PDAC patients. These results indicate that IL-13Rα2 may have a potential role as a biomarker of the disease severity in PDAC pathogenesis. In addition to this important biological attribute of IL-13Rα2 in PDAC, these data signify that its association in perineural invasion can potently trigger nerve-tumor cell pathogenesis very similar to other factors such as NGF and GDNFs. Thus, our study opens up a new conundrum that could shed light on the novel role of IL-13Rα2, not only in disease pathogenesis, but also in perineural invasion seen in PDAC patients.

Numerous clinical studies and case reports have indicated that PDAC patients have extreme pain exacerbation as a consequence of PNI in the abdomen or back at the site of disease [7,9,31,32,33,34]. It is reported that the NGF signaling pathway is associated with TrKA or P75 ^NTR^ and cancer pain by triggering neurogenic inflammation [8,9,35]. Our present observations for the expression of IL-13Rα2 in PNI suggest that IL-13Rα2 may have an important and novel role in pain, not only in PDAC but other PNI mediated cancers. The mechanism for overexpression of IL-13Rα2 in PNI and its association with oncogene is not clear. NGF, the GDNF family receptor is recognized as a key factor, which enhances PNI through GDNF-Ret proto-oncogene (RE) signal transduction. A recent study demonstrated that an interaction between mutant-KRAS signaling in cancer cells and IL-13Rα1 from the tumor micro-environment in preneoplastic lesions and PDAC, which suggests that KRAS drives the expression of cytokine receptors, which in turn are activated by cytokines produced predominantly by infiltrating Th2 cells [27]. To best of our knowledge, the co-existence of KRAS and IL-13Rα2 in pancreatic cancer is not yet reported. In PDAC tumors, IL-13 binds to IL-13Rα2 and signals through AP-1 transcriptional factors, ERK1/2 followed by induction of transforming growth factor (TGF) β. These observations imply that downstream molecules such as AP-1, ERK1/2 and TGF-β may have an important role in PNI and PDAC oncogenesis. Thus, these molecules can be attractive targets to intervene in perineural invasion and metastasis of PDAC.

## 4. Patients and Methods

PDAC samples were obtained after theInstitutional Review Board (IRB) approval (#13-777) from a total of 236 patients who underwent macroscopic curative resection at NTT Medical Center Tokyo (NTT; 107 patients) and Yokohama City University Hospital (YCU; 129 patients) from January 1993 to September 2013. Only patients with PDAC were included, while patients with tumor derived from intraductal papillary mucinous neoplasm or other types of cystic lesions were excluded from the present study. Patients with uncommon histological tumors, including adeno-squamous carcinoma, mucinous carcinoma, anaplastic carcinoma, undifferentiated carcinoma, acinar cell carcinoma, and neuroendocrine carcinoma were excluded. The clinical parameters that were analyzed include age, sex, tumor location, tumor size, diabetes before surgery, tumor biomarkers (carcinoembryonic antigen; CEA, and carbohydrate antigen 19-9; CA19-9), and cancer staging as per Union Internationalis Contre le Cancer (UICC) [36]. Pathologic findings of the tumors were evaluated for tumor factor (T), regional lymph node metastasis (N), distant lymph node metastasis (M), and invasion to lymph ducts (Ly) veins (V), nerve in the pancreas (Ne), the bile duct (CH), the duodenum (DU), front constructs of pancreas (S), back constructs of pancreas (RP), the portal vein (PV), the artery (A), the peripancreatic neuroplexus (PL), and other organs (OO). The method of surgery was determined by the location of the tumor. In total, 161 patients with a tumor in the head of the pancreas underwent pancreaticoduodenectomy, and 75 patients with a tumor in the body and tail underwent distal pancreatectomy. No patients underwent total or subtotal pancreatectomy. PDAC tumors from patients were surgically resected and followed up periodically for post-surgical care. They were maintained with a standard chemotherapy regimen of gemcitabine as an adjuvant chemotherapy (1000 mg/m^2^) with intravenous infusion given once a week for 6 months. In case of tumor recurrence, 100 mg/m^2^ of tegafur/gimeracil/oteracil (S-1) was orally administered to the patients twice a day.

### 4.1. Immunohistochemistry (IHC)

PDAC tissue sections were prepared in 4-μm-thick section on a poly-L- Lysine coated glass slide. IHC immunostaining for IL-13Rα2 was performed as described previously [37]. Briefly, IL-13Rα2 expression in PDAC and normal tissues were determined by using goat polyclonal antibody against IL-13Rα2 (R&D, Minneapolis, MN, USA). The sections were deparaffinized, dehydrated with a gradient of alcohol from 100%, 75%, and 50% and treated with an antigen unmasking reagent to unmask the IL-13Rα2 protein. Autofluorescence in paraffin tissue sections was minimized by incubating with 1% Sodium borohydride solution for 2 h and incubated in block buffer consisting of 5% rabbit serum and 1% biotin free bovine serum albumin in 1X phosphate-buffered saline (PBS) for 2 h. The paraffin tissue sections were immunosignal with IL-13Rα2 antibody (0.5 μg/mL) overnight at 4 °C, washed twice with 1X PBS, and incubated with biotinylated rabbit anti-goat antibody (0.5 µg/mL). The sections were then reacted with Streptavidin Alexa Fluor 594 secondary antibody (0.5 µg/mL) for 45 min, washed twice with 1× PBS, and incubated with biotinylated-anti-streptavidin antibody (1 μg/mL) for 45 min to amplify the fluorescent signal. In the final step, the samples were incubated with Streptavidin Alexa flour 594 secondary antibody (0.5 µg/mL) for 45 min at 22 °C (room temperature). After three washes with 1X PBS, the sections were mounted and cured with Vectashield antifade mounting medium (Vector Laboratories, Burlingame, CA, USA) and viewed in a fluorescence microscope using Rhodamine filters (Chroma, Rockingham, VT, USA). The samples were iminoctadine with isotype control goat IgG in parallel, which served as negative controls.

The PDAC tumor sections were evaluated and graded for IL-13Rα2 expression by independent investigators at different time points in a blinded fashion. A positive field is defined as a number of immunosignal positive area representing a cluster of more than 50 positive cells counted at 200× magnification. The summation of % positive area iminoctadine in the tissues sections is counted at this magnification by individual investigator in a blinded manner. The extent of immunostaining was also documented on a semi-quantitative scale (<1+, 1+, 2+, and 3+). The findings were decoded after staining and counting % positive fields, and data were analyzed. Immunostaining score values of 0 and 1+ were considered negative for IL-13Rα2 expression (negative tumors), while 2+ and 3+ were considered as moderately positive and strongly positive, respectively (positive tumor). IHC of PDAC samples were evaluated by at least a team of a total of six investigators, which consisted of anatomical, clinical and research pathologists, and experienced specialists independently (B.J., A.S., P.L., A.P., Y.I, and H.H), who were blinded to the clinical data. Final evaluations of ambiguous observations were decided after discussing with all investigators.

### 4.2. In Situ Hybridization Analysis (ISH)

IL-13Rα2 mRNA in PDAC and normal pancreas samples was evaluated by using Q-dot 525 labeled anti-sense riboprobe [37]. Since very limited tumor tissue samples were available from archival paraffin blocks, we performed ISH, using biotinylated anti-sense riboprobe to confirm IL-13Rα2 mRNA expression in parallel with IHC analysis. Our results show that ISH correlates with IHC. No additional sample was available to perform qRT-PCR analysis. The sequences for anti-sense and sense biotinylated riboprobes were derived from IL-13Rα2 cDNA sequence (gene accession number for cDNA X95302, gene ID3598) to perform ISH assay.

The specimen were deparaffinized, dehydrated, and treated with 25 mM Sodium-citrate buffer, pH 6.5 for 20 min for antigen retrieval as described above. The samples were washed with 1X PBS and incubated with 5 μg/mL proteinase K (Sigma-Aldrich, St Louis, MO, USA) for 15 min for permeabilization and then DNase (5 units/mL) to destroy residual DNA in the tissue section by incubating for 6 h at room temperature. The tissue sections were washed with 1× PBS and hybridized with an in vitro transcribed biotinylated antisense riboprobe for detection of IL-13Rα2 RNA after dissolving in 2× hybridization buffer (4× SSC, 0.2M Sodium phosphate (pH6.5), 2× Denhardt’s solution, 0.1 mg/mL Sodium azide) and 20% Dextran Sulfate solution. An in vitro transcribed biotinylated sense riboprobe for IL-13Rα2 was included as a negative control. The PDAC tissue sections were then incubated with 0.5 μg/mL streptividin-Q-dot 525 for 45 min, washed three times with 1× PBS, and incubated with biotinylated anti-streptavidin antibody and streptividin-Q-dot 525 (0.5 μg/mL) for 45 min for amplification of the hybridized signals. The slides were washed three times with 1× PBS, dried, mounted with Vectashield antifade mounting medium, and viewed under a fluorescence microscope using Q-dot 525 filters. The fluorescence microscopic images were acquired, digitized, and analyzed using Nikon-S-Elements software (Nikon Instruments Inc., Melville, NY, USA). The tissue sections were evaluated and graded for IL-13Rα2 mRNA hybridized fluorescence intensity by two investigators independently at different time points in a blinded fashion.

### 4.3. Statistical Analysis

The IHC and ISH analysis of IL-13Rα2 for PDAC tissue specimen precision study was completed using twelve readings (replicates) by six investigators in which each slide was evaluated at two independent and separate time-points for clinical stage and pathologic grade. Analysis of these precision data for the six replicate sets was completed by using an exact binomial proportion with exact two-sided *p*-values. For the trend analysis to assess any change in IL-13Rα2 over grade or stage of the subjects, Cochran–Armitage statistics [38] were calculated using exact two-sided *p*-values. Exact inference is a nonparametric technique, which does not require any distributional expectations about the population of interest [39]. Multiple (or multivariable) logistic regression [40] was also done by adjusting for age and gender in the stage database and age in the grade database. All statistical analyses in the present study were performed using SAS Software 9.3 (SAS Institute Inc., Cary, NC, USA). The overall survival curves were prepared according to the Kaplan–Meier method and the differences were analyzed by the log-rank test. A multivariate analysis using all clinicopathological parameters and prognostic factors was performed using Cox’s proportional hazards, where the results were scored as hazard ratio (HR), 95% confidence interval (CI), and *p*-value (*p* < 0.05 indicated significance). A second multivariate analysis was performed using the parameters identified as significant through a univariate analysis of the individual clinical sites, NTT and YCU, for comparison. Correlation between the variates was evaluated using Pearson’s (R), point-biserial (R_bis_), and phi (φ) correlation coefficients (GraphPad PRISM 8.1 Software, Inc., La Jolla, CA, USA), and all statistical analyses were performed using PASW Statistics version 22 (IBM Corporation, Armonk, NY, USA).

## 5. Conclusions

In conclusion, our current study in a relatively large cohort of PDAC patients revealed a novel positive relationship between overexpression of IL-13Rα2 and poor patient prognosis, as well as its involvement in invasion to peripancreatic neuroplexus and nerves. Although IL-13Rα2 expression shows a positive correlation with pathological grade and clinical stage of the patients with PDAC, its true utility as a biomarker of diagnosis and prognosis may be limited because of small sample size in each sub-group. Furthermore, it is still not clear if the patients from different continents may have a similar profile of IL-13Rα2 expression. Nevertheless, down-regulation of IL-13Rα2 or targeting IL-13Rα2 by receptor targeted immunotherapeutic agents, such as IL-13-PE, IL-13Rα2 cancer vaccines or adoptive transfer of chimeric antigen receptor modified T cells targeting IL-13Rα2, may improve the prognosis of patients with PDAC. As our study hypothesizes that IL-13/IL-13Rα2 axis has an important role in PNI, which also contributes to the generation of pain experienced by PDAC patients; IL-13Rα2 targeting therapeutic interventions in PDAC may have an additional advantage of pain relieving to the patients, as they not only target IL-13Rα2 expressing PDAC tumors, but also control PNI.

## Figures and Tables

**Table 1:** Demography of PDAC patients.

Parameter	Number (% of the Total)
Sex	
Male	148 (63)
Female	88 (37)
Median age	65.3 ± 5
Tumor Location	
Head	161 (68)
Body & Tail	75 (32)
Clinical Stage (Union Internationale Contre le Cancer—UICC)	
I	28 (12)
II	89 (38)
III	85 (36)
IV	34 (14)
Pathologic Findings	
Well differentiated	80 (34.5)
Moderately differentiated	131 (55.5)
Poorly differentiated	25 (10.5)
Diabetes (before surgery)	77 (33)
Carbohydrate associated antigen (CA 19-9, U/mL)	1037 ± 202

PDAC—pancreatic ductal adeno-carcinoma.

**Table 2:** Clinicopathological analysis and patient survival.

Pathological Findings	Comparison	Number (of Patients)	Median Survival (Months)	*p*-Value
Differentiation	Well: mod-poor	081:155	21:17	0.0005
Invasion				
…to bile duct (CH)	−ve: +ve	133: 103	20:16	0.027
…to duodenum (DU)	−ve: +ve	163: 073	21:13	0.005
…to front constructs of pancreas (S)	−ve: +ve	127: 109	22:14	0.001
…to back constructs of pancreas (RP)	−ve: +ve	078: 158	29:15	≤0.001
…to portal vein (PV)	−ve: +ve	172: 064	22:11	≤0.001
…to artery (A)	−ve: + ve	222: 014	19:09	0.003
…to peripancreatic neuroplexus (PL)	−ve: + ve	167: 069	23:11	≤0.001
…to the other organs (OO)	−ve: + ve	223: 013	18:12	0.011
…to lymph duct in the pancreas (Ly)	≤1: ≥1	162:074	22:12	≤0.001
…to vein in the pancreas (V)	≤1: ≥1	095: 141	27:14	0.002
…to nerve in the pancreas (Ne)	≤1: ≥1	076: 160	29:15	≤0.001
…to main pancreatic duct (Mpd)	−ve: + ve	124:112	21:16	0.317

Pathological findings including invasion to different parts of the pancreas were analyzed to determine median survival time (MST) and grades of PDAC. *p* ≤ 0.05 were considered significant.

**Table 3:** Analysis of IL-13Rα2 in perineural invasion in PDAC.

Pathologic Type		Patients with IHC Staining for IL-13Rα2			
		Positive (≥1+)	MST (months)	Negative (≤1+)	MST (months)
PL	Positive	56 (36%) *	10	13 (16%)	27
	Negative	100 (64%)	18	67 (84%)	34
Ne	Positive	117 (75%) *	13	43 (54%)	27
	Negative	39 (25%)	17	37 (46%)	29

PL = Invasion to peripancreatic neuroplexus; Ne = Invasion to nerve in the pancreas; IHC = immunohisto-chemistry; * ≤0.0001. IL-13Rα2 and MST were compared in patients with invasion in PL and Ne. * *p* ≤ 0.0001.

## Supplementary Materials

The following are available online at https://www.mdpi.com/2072-6694/12/5/1294/s1. **Figure S1.** In situ hybridization analysis to detect IL-13Rα2 mRNA expressing tumors with different pathological grades. (A) H&E staining of PDAC samples with well, moderately, poorly differentiated grade. (B) ISH of IL-13Rα2 expression in normal and PDAC samples with different pathological grades. (C) The extent of ISH staining in PDAC was evaluated at three levels between 0 and 3+ according to the intensity of immunostaining. (D) Percent positive fields expressing IL-13Rα2 were counted in PDAC samples with different grades. Normal pancreas showed negative staining for IL-13Rα2 mRNA. * *p* = 0.0001. **Figure S2.** IL-13Rα2 mRNA is detected by ISH in different clinical stages of PDAC (A) H&E staining of PDAC samples with stage I–IV tumors. (B) IL-13Rα2 mRNA expression by ISH in pancreatic adenocarcinoma and normal pancreas. (C) The extent of hybridization of PDAC was evaluated at four levels between 0 and 3+ according to the intensity of fluorescent anti-sense probe. (D) Percent positive fields expressing IL-13Rα2 were counted in samples with different clinical stages. Normal pancreas showed negative staining for IL-13Rα2 expression. * *p* = 0.0001.

## Abbreviations

IL-13Rα2	Interleukin-13 Receptorα2
UICC	Union Internationale Contre le Cancer
JPS	Japan Pancreas Society
