# Supplementary figures and images for: A Novel Role of Interleukin 13 Receptor alpha2 in Perineural Invasion and its Association with Poor Prognosis of Patients with Pancreatic Ductal Adenocarcinoma
Source: Cancers (Basel). 2020 May 20;12(5):1294. doi: 10.3390/cancers12051294 (PMC7281570; doi:10.3390/cancers12051294)

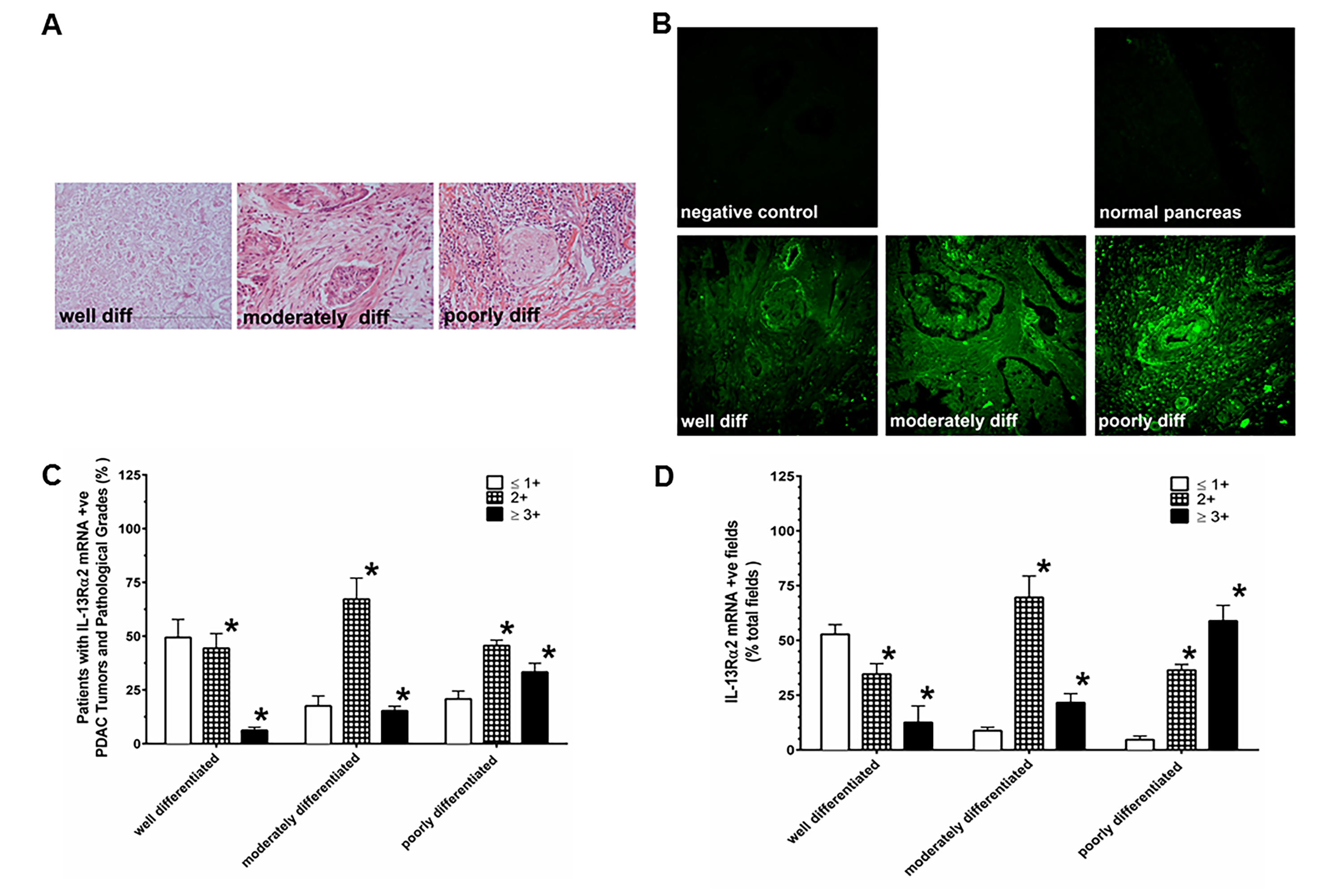

Supplement: Supplementary file 1 [file cancers-12-01294-s001.zip › SI Fig 1.TIF]

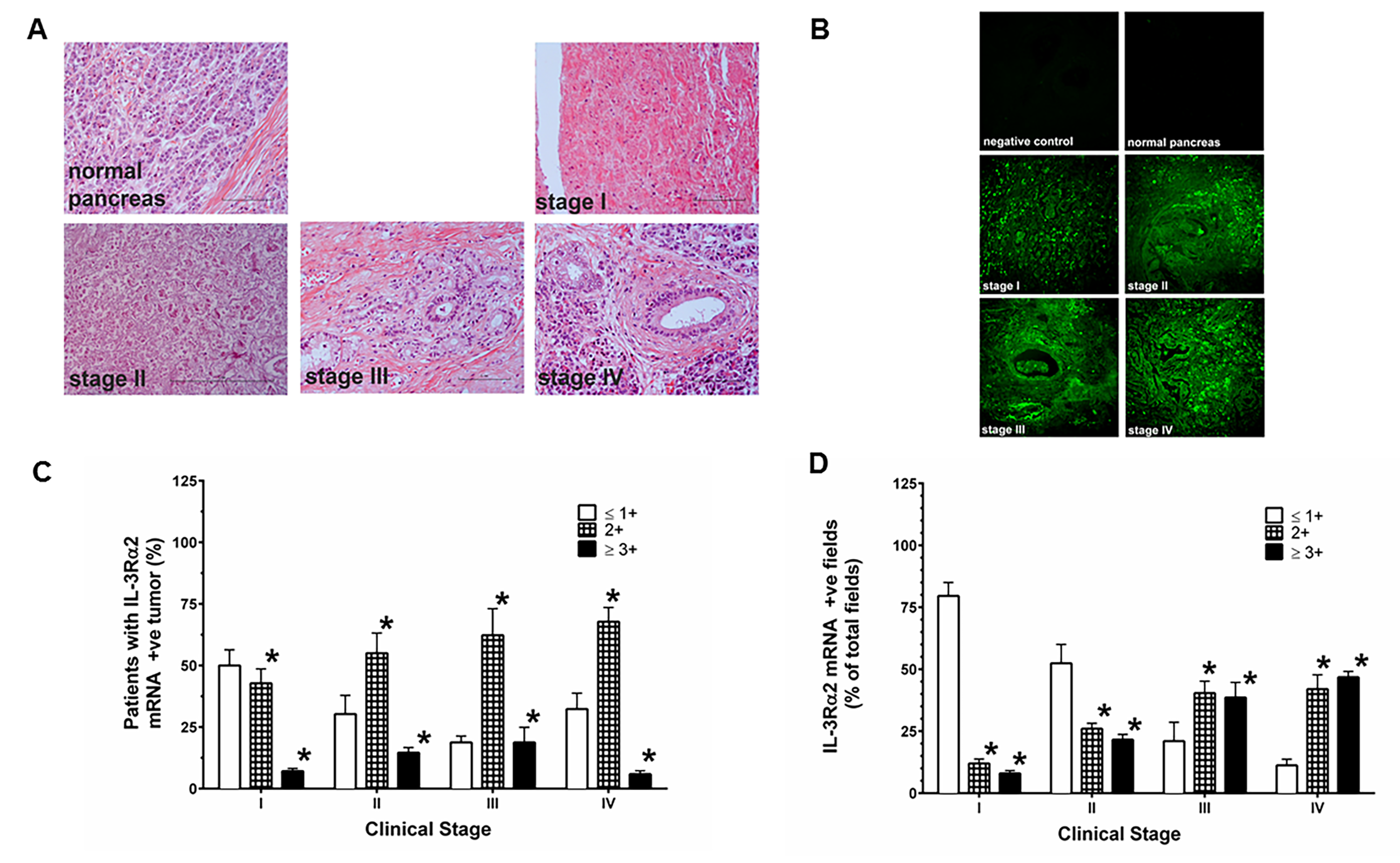

Supplement: Supplementary file 1 [file cancers-12-01294-s001.zip › SI Fig 2.TIF]
